# Puerperal Convulsions

**Published:** 1860-05

**Authors:** 


*Puerperal Convulsions.*—In the *New York Monthly Review &c.*, for January, Prof. White, of Buffalo, reports a case of puerperal convulsions of great severity, treated without blood-letting, but quite successfully, with chloroform, &c. "Dr. White thinks puerperal convulsions a disease *sui generis*, not apoplectic, nor epileptic; hence, bleeding is seldom necessary." * * * "The puerperal convulsions is, no doubt, caused by some remote uterine irritation, perhaps uræmia, though the latter is not constant. This condition tends to develop convulsions. His theory is, that we should give chloroform and anodynes to relieve this irritable state of the system, to be followed by croton oil as a counter-irritant. This is easily administered, and acts as a powerful revulsive. He thought we should never arrive at a correct theory, or satisfactory treatment of this disease, until we change our notions in relation to the character of the seizures." We copy the above opinions with pleasure, corresponding, as they do, exactly with our own. We believe more women have died when in child-bed from the lancet than from convulsions. Cases of puerperal convulsions probably do occur, requiring the abstraction of blood; upon this point we will not call in question the universal judgment of the profession, but we may be permitted to say that such we have never seen. In eleven years' experience we have never bled a case of puerperal convulsions, nor lost a woman in childbed. Eschewing the charge of egotism, we attribute this favorable issue rather to the casualties of good luck than to legitimate sequences of peculiarities of treatment.

